# High-Quality Biodiesel Production from Buriti (*Mauritia flexuosa*) Oil Soapstock

**DOI:** 10.3390/molecules24010094

**Published:** 2018-12-28

**Authors:** Samantha Siqueira Pantoja, Vanessa Albuquerque de Mescouto, Carlos Emmerson Ferreira da Costa, José Roberto Zamian, Geraldo Narciso da Rocha Filho, Luís Adriano Santos do Nascimento

**Affiliations:** 1 Laboratory of Catalysis and Oil-Chemistry, Graduate Program in Chemistry, Institute of Exact and Natural Sciences, Federal University of Pará, Belém, Pará, CEP 66075-110, Brazil; samanthasiqueira08@yahoo.com.br (S.S.P.); emmerson@ufpa.br (C.E.F.d.C.); zamian@ufpa.br (J.R.Z.); narciso@ufpa.br (G.N.d.R.F.); 2 Laboratory of Oils of the Amazon, Institute of Biological Sciences, Graduate Program in Biotechnology, Federal University of Pará, Belém, Pará, CEP 66075-110, Brazil; vanessadmescouto@gmail.com

**Keywords:** vegetable oil, esterification, biodiesel, waste valorization, *Mauritia flexuosa*

## Abstract

The buriti palm (*Mauritia flexuosa*) is a palm tree widely distributed throughout tropical South America. The oil extracted from the fruits of this palm tree is rich in natural antioxidants. The by-products obtained from the buriti palm have social and economic importance as well, hence the interest in adding value to the residue left from refining this oil to obtain biofuel. The process of methyl esters production from the buriti oil soapstock was optimized considering acidulation and esterification. The effect of the molar ratio of sulfuric acid (H_2_SO_4_) to soapstock in the range from 0.6 to 1.0 and the reaction time (30–90 min) were analyzed. The best conditions for acidulation were molar ratio 0.8 and reaction time of 60 min. Next, the esterification of the fatty acids obtained was performed using methanol and H_2_SO_4_ as catalyst. The effects of the molar ratio (9:1–27:1), percentage of catalyst (2–6%) and reaction time (1–14 h) were investigated. The best reaction conditions were: 18:1 molar ratio, 4% catalyst and 14 h reaction time, which resulted in a yield of 92% and a conversion of 99.9%. All the key biodiesel physicochemical characterizations were within the parameters established by the Brazilian standard. The biodiesel obtained presented high ester content (96.6%) and oxidative stability (16.1 h).

## 1. Introduction

Biodiesel is a fuel derived from vegetable oils or animal fat that is formed by mono-alkyl-esters of long chain fatty acids. It can be used for replacing fossil diesel fuel in whole or in part in compression ignition engines for automotive propulsion or energy generation [1]. One of the advantages of using biodiesel is the high cetane number compared to diesel. Biodiesel has no aromatics or sulfur, and it is composed of about 10 to 11% oxygen by weight. Consequently, the use of this fuel reduces the emissions of carbon monoxide, unburned hydrocarbons, and particulate matter when compared to diesel fuel. Furthermore, it is a renewable, biodegradable, alternative and nontoxic fuel [2].

There are several critical issues for biodiesel production, such as high prices and the low availability of raw vegetable oils. In addition, the high cost of feedstocks makes biodiesel production cost about 1.5 times more than diesel [3]. On the other hand, technological solutions have been proposed, such as using less pure raw materials such as frying oil, soapstocks and waste oil [4].

**Figure 1:**
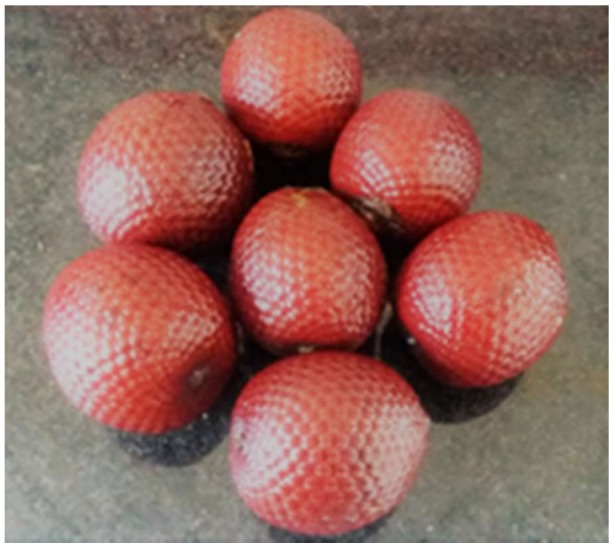
Buriti (*Mauritia flexuosa*) fruits.

There are approximately 4000 vegetable species around the world from which one can extract oil [5]. In Brazil, the Amazon region is known for its biodiversity, and has several industries that specialize in the production of vegetable oils, such as: açaí oil (*Euterpe oleracea*), andiroba oil (*Carapa guianensis*), buriti oil (*Mauritia flexuosa*), Brazil nut oil (*Bertholletia excelsa*), and copaiba oil (*Copaífera officinalis*). These oils are mainly used in the cosmetic, pharmaceutical and food industries. *Mauritia flexuosa* is one of the most common palms across tropical South America. The so-called “varzea” (floodplain) forests, in the Brazilian Amazonia, are largely dominated by *Mauritia flexuosa* and other palms [6]. The fruits of buriti (*Mauritia flexuosa*) (Figure 1) have a red and squamous shell, and a reddish and oily pulp with between 20–30% (wt.) of oil which has an elevated concentration of carotenes. It is estimated that the average annual production of pulp is approximately 0.79 t ha^−1^ and oil is 17.0 kg ha^−1^ [7].

Buriti oil is used in drugs, cosmetics, foods and polymeric materials. The search for new natural sources of β-carotene has encouraged the development of processes for extracting oil from buriti, which is rich in carotenoids [8,9]. Due to the presence of these natural antioxidants, this oil becomes less susceptible to oxidation; published studies have shown that buriti oil has a much higher oxidative stability (4155.6 min) than passion fruit oil (160.8 min) and rubber seed oil (291.6 min), measured in a Rancimat apparatus at 100 °C, suggesting that buriti oil could be useful in the food industry [10].

The chemical and physical refining processes of vegetable oils, such as degumming, neutralization, bleaching and deodorization, have been designed to remove substances that may impart undesirable flavor, color, or keeping quality and to obtain odorless, bland and oxidatively stable oils, which are acceptable to consumers [11]. About 6% of soapstock is generated during the process of refining vegetable oils. The cost of this material is around one-tenth of the price of refined oil, so it is a cheap source of fatty acids [12]. 

Soapstock consists of a heavy aqueous lipid emulsion having about 50% water, phosphatides, pigments and other minor non-polar compounds. In addition to these, it has salts of free fatty acids and triglycerides, which can be converted to biodiesel [13].

Generally, in the industrial processing of this material, acidification is first carried out, using sulfuric acid and steam to achieve partial acid hydrolysis and/or removal of the acyl and phosphoacylglycerol ester bonds of the starting material. After acidulation, the spontaneous separation into two phases occurs, an aqueous phase, and an oil layer, formed by pigments, acylglycerol-free fatty acids, and other lipophilic materials; this is called acid oil [14].

The main contribution reported in this paper was to test the best reaction conditions of the acidulation stages for soapstock and for esterification of acid oil in order to obtain and provide a previously unpublished physical and chemical description of the biodiesel produced, from waste derived from refining buriti oil, demonstrating its viability as a raw material for biofuel.

## 2. Results

### 2.1. Main Characteristics of Buriti Oil Soapstock

The main characteristics of the buriti oil soapstock are shown in Table 1. The acidity index was 6.9 mg of KOH/g. The moisture and volatiles were 9.1%. The fatty acids found in the soapstock were palmitic, palmitoleic, stearic, oleic, linoleic and linolenic acids, with the largest share being oleic acid (71.4%). Comparing the fatty acid composition of buriti oil found in the literature [7] with that of the soapstock used in this research, it was found that the compositions were similar.

### 2.2. Acidulation of the Buriti Oil Soapstock

#### Effect of the Acid/Soapstock Molar Ratio and Effect of the Time on Soapstock Acidulation

For the acidulation stage of the soapstock, molar ratios for sulfuric acid/soapstock of 0.6, 0.8 and 1.0 were used and the time was kept constant at 1 h. As shown in Table 2, when the molar ratio was increased from 0.6 to 0.8 there was an increase in conversion into fatty acids from 91.3% to 94.4%, while at a molar ratio of 1.0 conversion was 92.5%. Increasing the percentage of catalyst raised viscosity in the reaction slurry, and therefore slowed the mass transfer of the reagents to and from the surface of the catalyst, decreasing the reaction rate [15]. The molar ratio of 0.8 was thus established as the best condition. 

To evaluate the influence of time on acidulation of the soapstock, reactions of 30, 60 and 90 min were performed and the acid/soapstock molar ratio was kept constant at 0.8. As shown in Table 2, when the reaction time was increased from 30 min to 60 min there was an increase in conversion into fatty acids from 90.2% to 94.4%, while at 90 min of reaction, conversion was 91%. This behavior can be explained because catalyst activity is slightly inhibited by the formation of water in the reaction mixture [16]. The best conditions for acidulation of buriti oil soapstock were therefore established as an acid/soapstock molar ratio 0.8 and a reaction time of 60 min.

### 2.3. Esterification of the Acid Oil

#### 2.3.1. Effect of Methanol/Oil Molar Ratio on Esterification of Acid Oil

The methanol/oil ratio is one of the important factors that affect conversion [12]. Different molar ratios 9:1, 18:1 and 27:1 were investigated, while the other conditions were kept constant (4% of H_2_SO_4_, 1 h). Theoretically, 1 mol of biodiesel is originated from 1 mol of alcohol and 1 mol of feedstocks. However, esterification is a reversible reaction, and thus requires a greater amount of alcohol for the reaction to proceed [17]. When the molar ratio was raised from 9:1 (77.3%) to 18:1 (83.9%), there was an increase in the ester content, but when the molar ratio 27:1 (72.4%) was used, it was found that the ester content was reduced; therefore, a molar ratio of 18:1 showed better performance. This behavior occurs because, when there is an excess of methanol, the reaction is slowed due to dilution of the reaction medium [18].

#### 2.3.2. Effect of the Percentage of Catalyst on Esterification of Acid Oil 

Different amounts of sulfuric acid, 2%, 4% and 6%, were investigated under the same conditions (molar ratio alcohol/acid oil 18:1; 1 h). As the percentage of catalyst was raised from 2% to 4%, there was a small increase in the ester content, although with 6% of catalyst there was a reduction in the ester content, so that 4% of H_2_SO_4_ was defined as the best condition. This result can be explained because the higher the amount of catalyst the greater the number of active sites, and consequently, the greater the conversion to free fatty acid (FFA) in a lower reaction time. However, the addition of a large amount of catalyst to the reaction medium increases the viscosity and decreases the surface contact between the active sites of the catalysts and the reactants [19].

#### 2.3.3. Effect of Time on Esterification of Acid Oil

**Figure 2:**
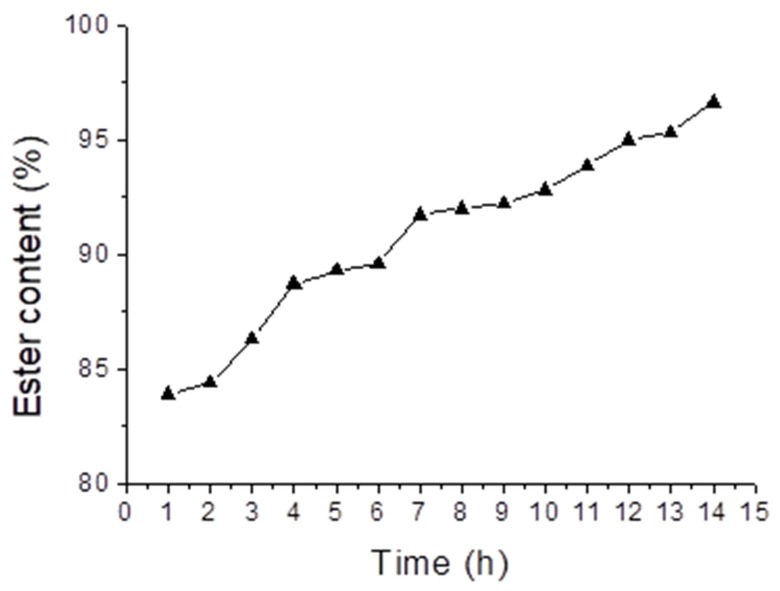
Effect the time on esterification of acid oil. Reaction conditions: Molar ratio of methanol/acid oil 18:1; 4% of H_2_SO_4_.

To evaluate the influence of time on esterification of acid, oil aliquots were taken every hour. As shown in Figure 2, during the first hour of the reaction, the ester level was 83.9%; at 6 h it reached a value of 89.6%, and only at 14 h was a value of 96.6% achieved, which is within the standards required by the National Agency for Petroleum, Natural Gas and Biofuels (ANP) [20] (min. 96.5%). Therefore, the reaction conditions that showed the best performance were a molecular ratio for alcohol/acid oil of 18:1, 4% of H_2_SO_4_ and 14 h reaction time. For comparison purposes, Guo et al. (2012) [21] synthesized biodiesel from acidified soybean soapstock using a lignin-derived carbonaceous catalyst, with conversion above 93.4% at 5 h reaction time.

### 2.4. Physical-Chemical Characterization of the Biodiesel Obtained from Buriti Oil Soapstock

The biodiesel yield obtained from buriti oil soapstock was 92%, calculated with respect to the initial mass of the acid oil used. Table 3 shows the results obtained with the physical-chemical characterization of the biodiesel from buriti oil soapstock, the limits stipulated in Brazil, according to the Resolution of the National Agency for Petroleum, Natural Gas and Biofuels (RANP 45/2014), European standard (EN 14214) and American standard (ASTM D 6751).

Specific mass for a biodiesel is related to its molecular structure. Specific mass is directly proportional to the carbonic chain size of the alkyl ester and inversely proportional to the number of double bonds [22]. The specific mass of the biodiesel derived from buriti oil soapstock was 877.3 kg/m^3^; a value within the limits specified in RANP 45/2014, this parameter influences the efficiency of fuel atomization [23].

The viscosity of biodiesel obtained from buriti oil soapstock was 5.22 mm^2^/s; that value is within the limits established by RANP 45/2014 and ASTM D 6751, and above that established by EN 14214. This characteristic is important because fuels with high viscosity tend to form larger droplets upon injection, resulting in poorer atomization and creating problems such as enhanced polymerization reaction and more carbon deposits. Additionally, a highly viscous fuel mixes more slowly with air and causes weak combustion, exhaust smoke and emissions, apart from problems caused by increased viscosity in cold weather. On the other hand, a fuel with low viscosity cannot provide sufficient lubrication for the accurate fit of fuel injection pumps, resulting in increased wear or leakage [24].

The minimum flash point required by the RANP is 100 °C, ASTM D6751 is 130 °C and EN 14214 is 101 °C. The flash point of biodiesel obtained from buriti oil soapstock was 174 °C. Flash point is a property related to safety that is usually considered in determining handling, transportation and fuel storage conditions. This property is defined as the minimum temperature at which the volatile fuel flashes or ignites momentarily when in contact with a flame or spark at a pressure of 101.325 kPa [25].

Acid value indicates the presence of free fatty acids in a fuel sample. Presence of free fatty acids in biodiesel may cause engine corrosion and the formation of deposits [26]. The acidity of biodiesel obtained from buriti oil soapstock was 0.04 mg KOH/g, well above the maximum value allowed by RANP norm 45/2014, ASTM D6751 and EN 14214 (0.5 mg KOH/g) indicating that this biofuel has a small amount of free fatty acids, since there was a high conversion of those esters in the esterification reaction. This would probably not cause problems in relation to engine corrosion.

Cold filter plugging point (CFPP) is measured using a standard device; it is the lowest temperature (°C) at which the biodiesel can cross this device during a specific time [27]. Cold filter plugging point (CFPP) of a fuel is related to performance in cold climates. With low operating temperature, fuel consumption can diminish and reduce the performance of fuel pumps, lines and injectors [28]. The CFPP for biodiesel obtained from buriti oil soapstock was 3 °C, a value below the maximum level stipulated in RANP 45/2014 and EN 14214, indicating that this fuel can be used without problems in cold weather regions. This value is related to the high content of esters of unsaturated acids in this biofuel, at around 77.2%.

Corrosivity to copper is a property established for determining the potential capacity a fuel has of causing corrosion in metal parts; high values of this property indicate that the fuel can cause corrosion problems in the engine and tank. This property is associated with the presence of acids or sulfur compounds [22]. Corrosivity to copper of biodiesel obtained from buriti oil soapstock was 1, which is the threshold value stipulated by RANP 45/2014 and EN 14214. And corrosivity to copper of biodiesel obtained from buriti oil soapstock is below the value stipulated in ASTM D 6751.

**Figure 3:**
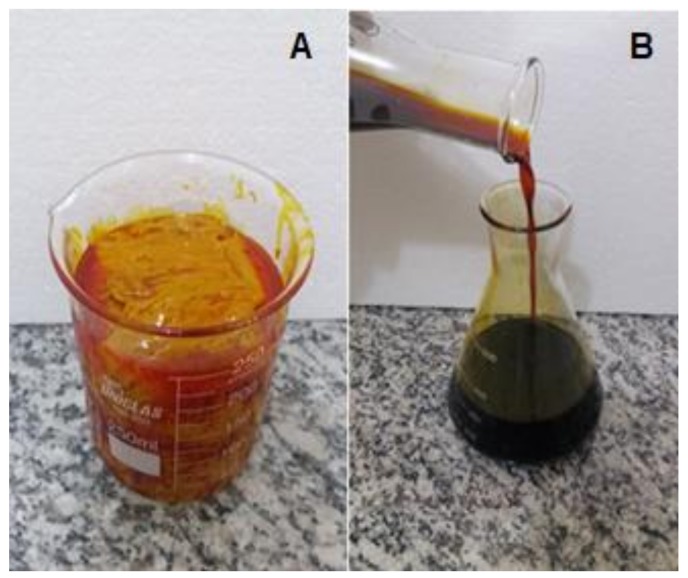
(**A**) Buriti oil soapstock and (**B**) biodiesel from buriti oil soapstock.

The biodiesel obtained presented a high level of esters, around 96.6%, indicating its elevated purity and the high efficiency of the esterification reaction. This value is above the minimum value of 96.5%, stipulated by RANP 45/2014 and EN 14214. Figure 3 shows (A) Buriti oil soapstock, a heavy emulsion and (B) Biodiesel from buriti oil soapstock, with a low viscosity.

The high oxidative stability of biodiesel from the buriti oil soapstock was 16.1 h; this value is above the minimum stipulated by RANP 45/2014 (8 h), ASTM D 6751 (3 h) and EN 14214 (6 h). Several researchers have studied the oxidative stability of biodiesel. During long periods of storage, this is one of the problems that affects biodiesel. Other parameters that influence stability, such as the presence of heat, air, light and peroxides, have been studied [29]. The high stability of this biodiesel may be influenced by the fatty acids’ composition of the soapstock, which presented mostly oleic acid (C18:1), around 71.4%, and palmitic acid (C16:0), around 21.1%. The relative rates of oxidation of the methyl esters of oleic (C18:1), linoleic (C18:2) and linolenic (C18:3) acids is 1:12:25 [30]. The presence of saturated fatty acid, which is less susceptible to oxidation, may also have contributed. Additionally, buriti oil has tocopherols and carotenoids, which are natural antioxidants, and provide this biodiesel with high oxidative stability [31].

## 3. Materials and Methods 

### 3.1. Materials

The soapstock from buriti oil was kindly donated by the Beraca Ingredientes Naturais S.A. company, located in the municipality of Ananindeua, in the State of Pará. The reagent used for acidification was sulfuric acid (95–97%) purchased from Fmaia (Belo Horizonte, Brazil). The reagents used in the characterization of the buriti oil soapstock, in the esterification of acid oil and in the biodiesel characterization were as follows: sodium hydroxide was obtained from Ecibra (São Paulo, Brazil), sulfuric acid (95–97%) was obtained from Fmaia (Fmaia, Brazil), sodium sulfate (99.9%) was purchased from Isofar (Duque de Caxias, Brazil), methyl alcohol (99.9%) and heptane were purchased from Dinamica (Diadema, Brazil), and analytical grade BF3 (12% in methanol) was obtained from Acros organics (Alvorada, Brazil). Analytical standards, MSTFA (*N*-methyl-*N*-trimethylsilyl trifluoroacetamide), methyl ester fatty acid (C4:0–C24:0) and C17:0 (methyl heptadecanoate (99.9%) were obtained from Aldrich (São Paulo, Brazil).

### 3.2. Characterization of the Buriti Oil Soapstock

The acidity index was determined according to AOCS Ca 5a-40. The moisture and volatiles were determined according to AOCS Ca 2c-25. The fatty acid composition of the soapstock was conducted using the standard method (AOCS Ce2-66), with a Varian CP 3800 gas chromatography system (São Paulo, Brazil). Capillary column Agilent (Santa Clara, CA, USA) (30 m × 0.32 mm CP WAX 52 CB; 1 µm DF) was used for the analysis. The column temperature was kept at 80 °C for 2 min, 180 °C for 1 min, and 250 °C for 5 min. Helium was used as the gas carrier (1.0 mL/min) and a hydrogen flame ionization detector was employed. The internal standard employed was methyl heptadecanoate.

### 3.3. Acidulation of the Soapstock

In order to optimize the acidulation stage of buriti oil soapstock several molar ratios of sulfuric acid/soapstock were used (0.6, 0.8 and 1.0) and the reaction time was kept constant (1 h). The effect of the reaction time for this stage was also evaluated (30, 60 and 90 min). Acidulation was performed using a heater blanket with agitation, a round-bottom balloon flask and a condenser. The soapstock was heated to a temperature of approximately 65 °C. The catalyst (H_2_SO_4_) was slowly added to the soapstock under intense agitation. This reaction mixture was kept under shaking at a temperature of approximately 90 °C. After the end of acidulation, the mixture was placed in a decantation funnel for 24 h, until separation of the phases occurred. The lower phase, containing free sulfuric acid, sodium sulfate and water-soluble impurities was discarded. The acid oil phase was washed three times using water at 90 °C. The acidity index of the product was determined in order to calculate the conversion later on.

The conversion of free fatty acids was calculated by the Equation (1):

Conversion (%) = [(a_i_ − a_f_)/a_i_] × 100 (1)

where a_i_ is the initial acidity of the soapstock and a_f_ is the final acidity, after acidulation.

### 3.4. Esterification of Acid Oil

The acid oil obtained was esterified using methanol and sulfuric acid as a catalyst. The acid oil was heated to approximately 50 °C, and the alcohol/catalyst mixture was then added, with the material kept under reflux conditions. The biodiesel obtained was washed in a decantation funnel with hot water in order to remove catalyst and alcohol residues. After washing, the material was dried with anhydrous sodium sulfate (4% *m*/*m*). To optimize esterification stage, different methanol/acid oil molar ratios were utilized (9:1, 18:1 and 27:1), and the other variables were kept constant (4% of H_2_SO_4_, 1 h). Next, reactions were performed with different percentages of catalyst (H_2_SO_4_) (2%, 4% and 6%) and molar ratio of methanol/acid oil and time remained constant (18:1; 1 h). Finally, the reaction time was evaluated (1–14 h), and molar ratio methanol/acid oil and percentage of catalyst remained constant (18:1; 4% of H_2_SO_4_). Aliquots of the reaction mixture were taken every hour, until the reaction reached the minimum ester content stipulated by the ANP [19]. The ester contents of the products were analyzed according to EN 14103, by gas chromatography (GC).

### 3.5. Properties of Methyl Esters

The properties of methyl esters were determined by standard methods: Specific mass, kinematic viscosity, flash point, copper corrosivity and cold filter plugging point were determined according to ASTM D 4052, 445, 93, 130, 6331, respectively. Acidity index was determined according to EN 14104. Oxidative stability was determined according to EN 14112, using the Rancimat Metrohm 743 (Herisau, Switzerland).

## 4. Conclusions

The biodiesel produced was of high quality, with all of the physical-chemical properties analyzed being within the limits established by the Brazilian, American and European standards, except for the viscosity that was above the value stipulated in EN 14214, which demonstrates the viability of buriti oil soapstock as a raw material for producing biofuel.

## Figures and Tables

**Table 1:** Main characteristics of the buriti soapstock oil and buriti oil.

	Soapstock	Buriti Oil ^1^
**Characteristics**		
Acid value (mg KOH/g oil) Moisture and volatiles (%)	6.9 9.1	
**Components fatty acids (wt% of total oil)**		
Palmitic acid (C16:0)	21.1	15.99
Palmitoleic acid (C16:1)	0.2	
Stearic acid (C18:0)	1.6	1.39
Oleic acid (C18:1)	71.4	77.06
Linoleic acid (C18:2)	2.4	1.58
Linolenic acid (C18:3)	3.2	1.12
Others		2.29
Average molecular weights of fatty acids	276.5	

^1^ Cunha et al., 2012 [7].

**Table 2:** Effect of the acid/soapstock molar ratio ^a^ and effect of the time on soapstock acidulation ^b^.

Molar Ratio of Acid/Soapstock ^a^	0.6	0.8	1.0
Conversion (%)	91.3	94.4	92.5
Time (min) ^b^	30	60	90
Conversion (%)	90.2	94.4	91

^a^ Reaction conditions: Time of reaction (1 h). ^b^ Reaction conditions: Molar ratio of acid/soapstock (0.8).

**Table 3:** Physical-chemical characterization of the biodiesel obtained from buriti oil soapstock.

Properties (Units)	RANP 45/2014	ASTMD 6751 **	EN 14214 ***	B100 Buriti Soapstock
Specific mass at 20 °C (kg/m^3^)	850–900	-	-	877.3
Kinematic viscosity at 40 °C (mm^2^/s)	3.0–6.0	1.9–6.0	3.5–5.0	5.22
Flash point min. (°C)	100	130	101	190
Acidity index max. (mg KOH/g)	0.5	0.5	0.5	0.04
Corrosivity to copper max.	1	3	1	1
Cold filter plugging point max. (°C)	19	-	−20	3
Ester content min. (% mass)	96.5	-	96.5	96.6
Oxidative stability min. (h)	8	3	6	16.5

** American Society for Testing and Materials; *** European standard.

## References

[1] Valente O.S., Pasa V.M.D., Belchior C.R.P., Sodré J.R. (2011). Physical-chemical properties of waste cooking oil biodiesel and castor oil biodiesel blends. Fuel.

[2] Zhang J., Zhang L., Jia L. (2011). Variables affecting biodiesel production from *Zanthoxylum bungeanum* seed oil with high free fatty acids. Ind. Eng. Chem. Res..

[3] Phan A.N., Phan T.M. (2008). Biodiesel production from waste cooking oils. Fuel.

[4] Marchetti J.M. (2012). A summary of the available technologies for biodiesel production based on a comparison of different feedstock’s properties. Process Saf. Environ. Prot..

[5] Santori G., Nicola G.D., Moglie M., Polonara F. (2012). A review analyzing the industrial biodiesel production practice starting from vegetable oil refining. Appl. Energy.

[6] Rull V., Montoya E. (2014). *Mauritia flexuosa* palm swamp communities: Natural or human-made? A palynological study of the Gran Sabana region (northern South America) within a neotropical context. Quat. Sci. Rev..

[7] Cunha M.A.E., Neves R.F., Souza J.N.S., França L.F., Araujo M.E., Brunner G., Machado N.T. (2012). Supercritical adsorption of buriti oil (*Mauritia flexuosa* Mart.) in γ-alumina: A methodology for the enriching of anti-oxidants. J. Supercrit. Fluids.

[8] Ribeiro R.D., Coelho M.A.Z., Barreto D.W. (2012). Production of concentrated natural beta-carotene from buriti (Mauritia vinifera) oil by enzymatic hydrolysis. Food Bioprod. Process..

[9] Aquino J.S., Pessoa D.C.N.P., Araújo K.L.G.V., Epaminondas P.S., Schuler A.R.P., Souza A.G., Stamford T.L.M. (2012). Refining of buriti oil (*Mauritia flexuosa*) originated from the Brazilian Cerrado: Physicochemical, thermal-oxidative and nutritional implications. J. Braz. Chem. Soc..

[10] Pardauil J.J.R., Souza L.K.C., Molfetta F.A., Zamian J.R., Rocha Filho G.N., Costa C.E.F. (2011). Determination of the oxidative stability by DSC of vegetable oils from the Amazonian area. Bioresour. Technol..

[11] Farhoosh R., Einafshar S., Sharayei P. (2009). The effect of commercial refining steps on the rancidity measures of soybean and canola oils. Food Chem..

[12] Su E., Wei D. (2014). Improvement in biodiesel production from soapstock oil by one-stage lipase catalyzed methanolysis. Energy Convers. Manag..

[13] Haas M.J., Bloomer S., Scott K.M. (2000). Simple, high-efficiency synthesis of fatty acid methyl esters from soapstock. J. Am. Oil Chem. Soc..

[14] Haas M.J., Michalski P.J., Runyon S., Nunez A., Scott K.M. (2003). Production of fame from acid oil, a by-product of vegetable oil refining. J. Am. Oil Chem. Soc..

[15] Piker A., Tabah B., Perkas N., Gedanken A. (2016). A green and low-cost room temperature biodiesel production method from waste oil using egg shells as catalyst. Fuel.

[16] Mekala M., Goli V.R. (2015). Kinetics of esterification of methanol and acetic acid with mineral homogeneous acid catalyst. Chin. J. Chem. Eng..

[17] Soltani S., Rashid U., Al-Resayes S.I., Nehdi I.A. (2017). Recent progress in synthesis and surface functionalization of mesoporous acidic heterogeneous catalysts for esterification of free fatty acid feedstocks: A review. Energy Convers. Manag..

[18] Shuit S.H., Tan S.H. (2014). Biodiesel production via esterification of palm fatty acid distillate using sulphonated multi-walled carbon nanotubes as a solid acid catalyst: Process study, catalyst reusability and kinetic study. BioEnergy Res..

[19] Xie W., Zhao L. (2014). Heterogeneous cao–moo3–sba-15 catalysts for biodiesel production from soybean oil. Energy Convers. Manag..

[20] Agência Nacional do Petróleo, Gás Natural e Biocombustíveis. http://www.anp.gov.br/?pg=76798/.

[21] Guo F., Xiu Z.-L., Liang Z.-X. (2012). Synthesis of biodiesel from acidified soybean soapstock using a lignin-derived carbonaceous catalyst. Appl. Energy.

[22] Lôbo I.P., Ferreira S.L.C., Cruz R.S. (2009). Da biodiesel: Parâmetros de qualidade e métodos analíticos. Quím. Nov..

[23] Saloua F., Saber C., Hedi Z. (2010). Methyl ester of [maclura pomifera (rafin.) schneider] seed oil: Biodiesel production and characterization. Bioresour. Technol..

[24] Freitas S.V.D., Pratas M.J., Ceriani R., Lima A.S., Coutinho J.A.P. (2011). Evaluation of predictive models for the viscosity of biodiesel. Energy Fuels.

[25] Sajjadi B., Raman A.A.A., Arandiyan H. (2016). A comprehensive review on properties of edible and non-edible vegetable oil-based biodiesel: Composition, specifications and prediction models. Renew. Sustain. Energy Rev..

[26] Kakati J., Gogoi T.K., Pakshirajan K. (2017). Production of biodiesel from Amari (Amoora Wallichii King) tree seeds using optimum process parameters and its characterization. Energy Convers. Manag..

[27] Patel A., Arora N., Mehtani J., Pruthi V., Pruthi P.A. (2017). Assessment of fuel properties on the basis of fatty acid profiles of oleaginous yeast for potential biodiesel production. Renew. Sustain. Energy Rev..

[28] Meher L.C., Sagar D.V., Naik S.N. (2006). Technical aspects of biodiesel production by transesterification—A review. Renew. Sustain. Energy Rev..

[29] Knothe G. (2005). Dependence of biodiesel fuel properties on the structure of fatty acid alkylesters. Fuel Process. Technol..

[30] Jain S., Sharma M.P. (2010). Stability of biodiesel and its blends: A review. Renew. Sustain. Energy Rev..

[31] Albuquerque M.L.S., Guedes I., Alcantara P., Moreira S.G.C. (2003). Infrared absorption spectra of Buriti (*Mauritia flexuosa* L.) oil. Vib. Spectrosc..

